# Clinical and Echocardiographic Spectrum of Ostium Secundum Atrial Septal Defects in Adults: A Case Series and Management Insights

**DOI:** 10.7759/cureus.109832

**Published:** 2026-05-28

**Authors:** Khaoula Aboubakr, Amal Baicha, Mohamed Malki, Iliyasse Asfalou, Aatif Benyass

**Affiliations:** 1 Cardiology Center, Military Hospital Mohamed V, Rabat, MAR

**Keywords:** ostium secundum atrial septal defect, percutaneous closure, surgical repair, transesophageal echocardiography, transthoracic echocardiography

## Abstract

Ostium secundum atrial septal defect (ASD) is the most common congenital heart malformation in adults. Clinical presentation and echocardiographic findings are highly variable, influencing management strategies and outcomes.

We present a case series of four adults with ostium secundum ASD at different stages of disease. All patients underwent comprehensive transthoracic echocardiography, completed by transesophageal echocardiography. Clinical, echocardiographic, and therapeutic data were analyzed to illustrate the spectrum of presentations and guide individualized management.

Echocardiography accurately assessed defect size, morphology, shunt significance, right heart remodeling, and pulmonary hemodynamics. Cases ranged from a young patient with an isolated, easily correctable defect to adults with large or anatomically challenging defects, including one with severe pulmonary hypertension and atrial fibrillation. Management varied accordingly: percutaneous device closure was performed in anatomically suitable patients, while surgical repair was indicated in complex or late-diagnosed cases. Conservative follow-up was appropriate for small, hemodynamically insignificant defects.

In conclusion, ostium secundum ASD in adults exhibits wide clinical and echocardiographic variability. Individualized evaluation using transthoracic echocardiography (TTE), transesophageal echocardiography (TEE), three-dimensional imaging, and intracardiac echocardiography is essential to optimize management. Early diagnosis and a patient-centered, anatomy-driven approach allow appropriate selection of percutaneous or surgical intervention, prevent complications, and improve long-term outcomes.

## Introduction

Atrial septal defect (ASD) is one of the most common congenital heart diseases, accounting for approximately 25%-30% of all congenital heart defects diagnosed in adulthood. It results from incomplete closure of the interatrial septum, creating an abnormal communication between the left and right atria. This communication leads to a left-to-right shunt - meaning that oxygenated blood flows back into the pulmonary circulation - whose direction and magnitude depend on defect size, the relative pressures in both circulations, and the ability of each ventricle to fill and relax (ventricular compliance). Among the anatomical subtypes, ostium secundum ASD is the most frequent, representing nearly 75% of all ASDs.

Clinical presentation varies widely, even among patients sharing the same anatomical subtype, ranging from asymptomatic incidental findings in young patients to significant right heart remodeling and pulmonary hypertension in adults with long-standing unrepaired defects. This heterogeneity poses a real clinical challenge in terms of timing and choice of intervention.

Despite a growing body of literature on ASD management, few studies have systematically compared patients with the same anatomical subtype across different stages of the disease. In this context, we present a comparative case series of four patients diagnosed with ostium secundum ASD, aiming to illustrate the broad spectrum of clinical and echocardiographic presentations and to highlight how these variations influence therapeutic decision-making. This series adds to the existing literature by emphasizing the critical role of age, defect size, and cardiac remodeling in determining the optimal timing and modality of intervention.

## Case presentation

Case 1

**Figure 1:**
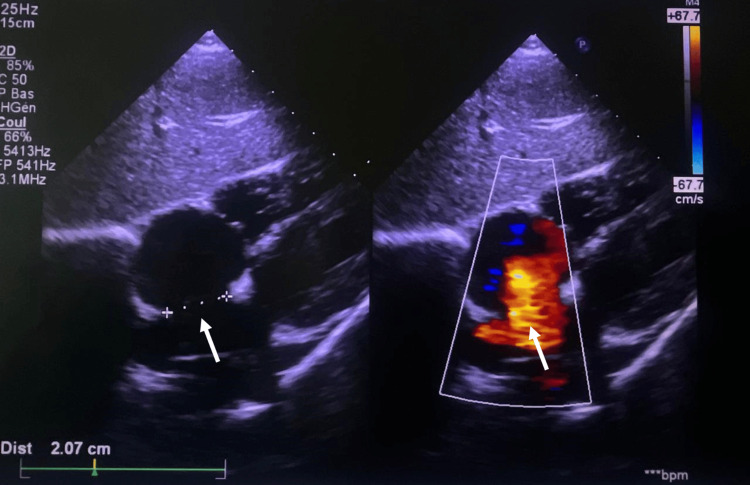
Transthoracic echocardiography (apical four-chamber view) of Case 1. The left panel shows a 2D image of the ostium secundum atrial septal defect measuring 20 mm (white arrow), with adequate septal rims. The right panel demonstrates color Doppler imaging confirming a significant left-to-right shunt (white arrow), consistent with a Qp/Qs ratio of 2.0 and systolic pulmonary artery pressure (SPAP) of 35 mmHg, supporting the decision for percutaneous closure.

A 17-year-old male presented with progressively worsening exertional dyspnea, leading to limitation of his usual sports activities. Physical examination revealed stable hemodynamics. Cardiac auscultation showed fixed splitting of the second heart sound over the pulmonary area, without associated murmurs. Electrocardiography was unremarkable. Transthoracic echocardiography (TTE) demonstrated an ostium secundum atrial septal defect (ASD) with a significant left-to-right shunt (Qp/Qs = 2) (Qp/Qs = pulmonary-to-systemic flow ratio), associated with mild tricuspid regurgitation, right ventricular dilation, and low probability of pulmonary hypertension. Transesophageal echocardiography (TEE) confirmed a defect measuring 20 × 27 × 25 mm with adequate septal rims suitable for device closure. After multidisciplinary discussion, the patient underwent successful percutaneous closure without complications (Figure 1).

Case 2

**Figure 2:**
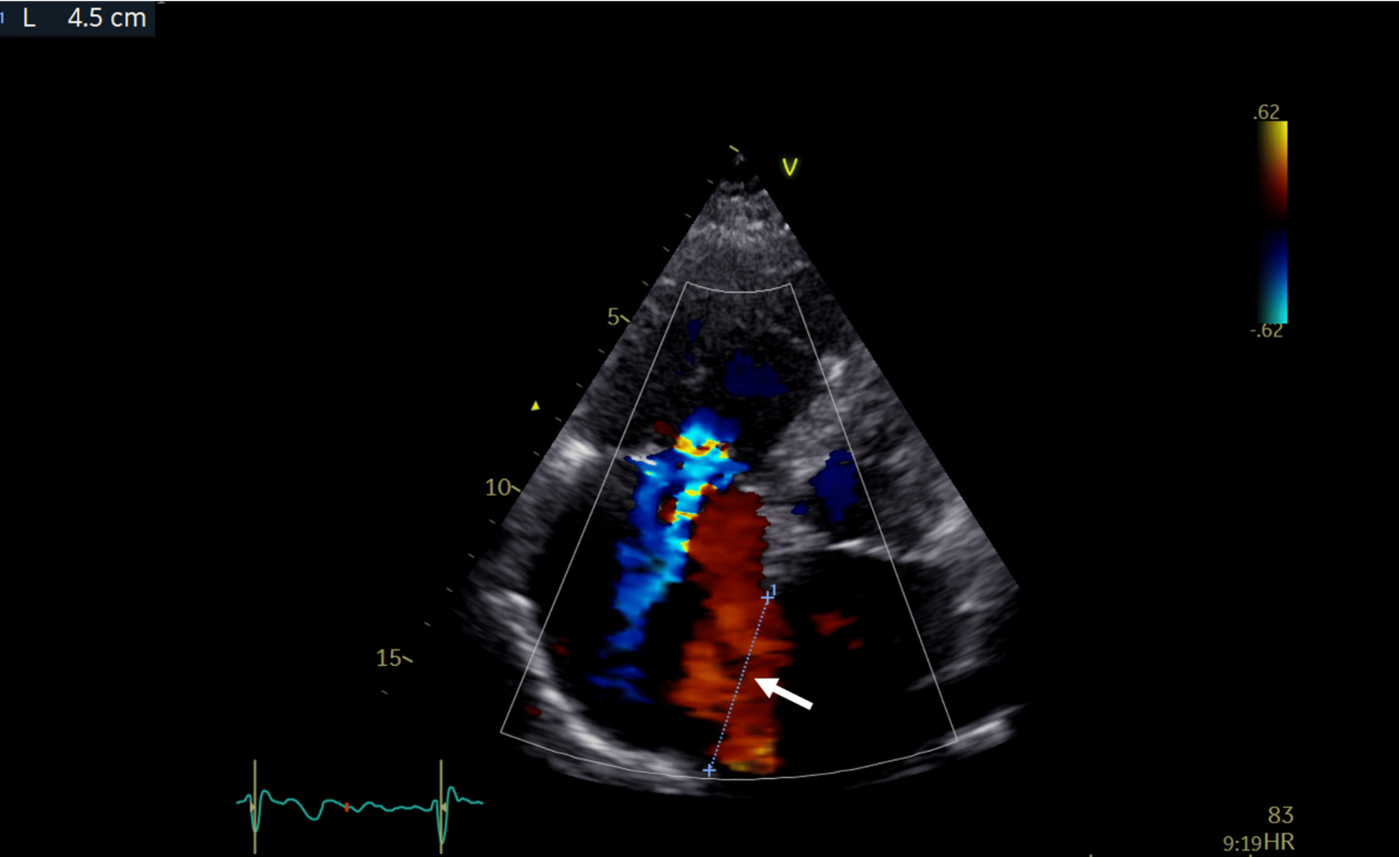
Color Doppler echocardiography (apical four-chamber view) of Case 2 showing a large ostium secundum atrial septal defect (ASD) measuring 45 mm with a significant left-to-right shunt (white arrow) and severe pulmonary hypertension.

**Figure 3:**
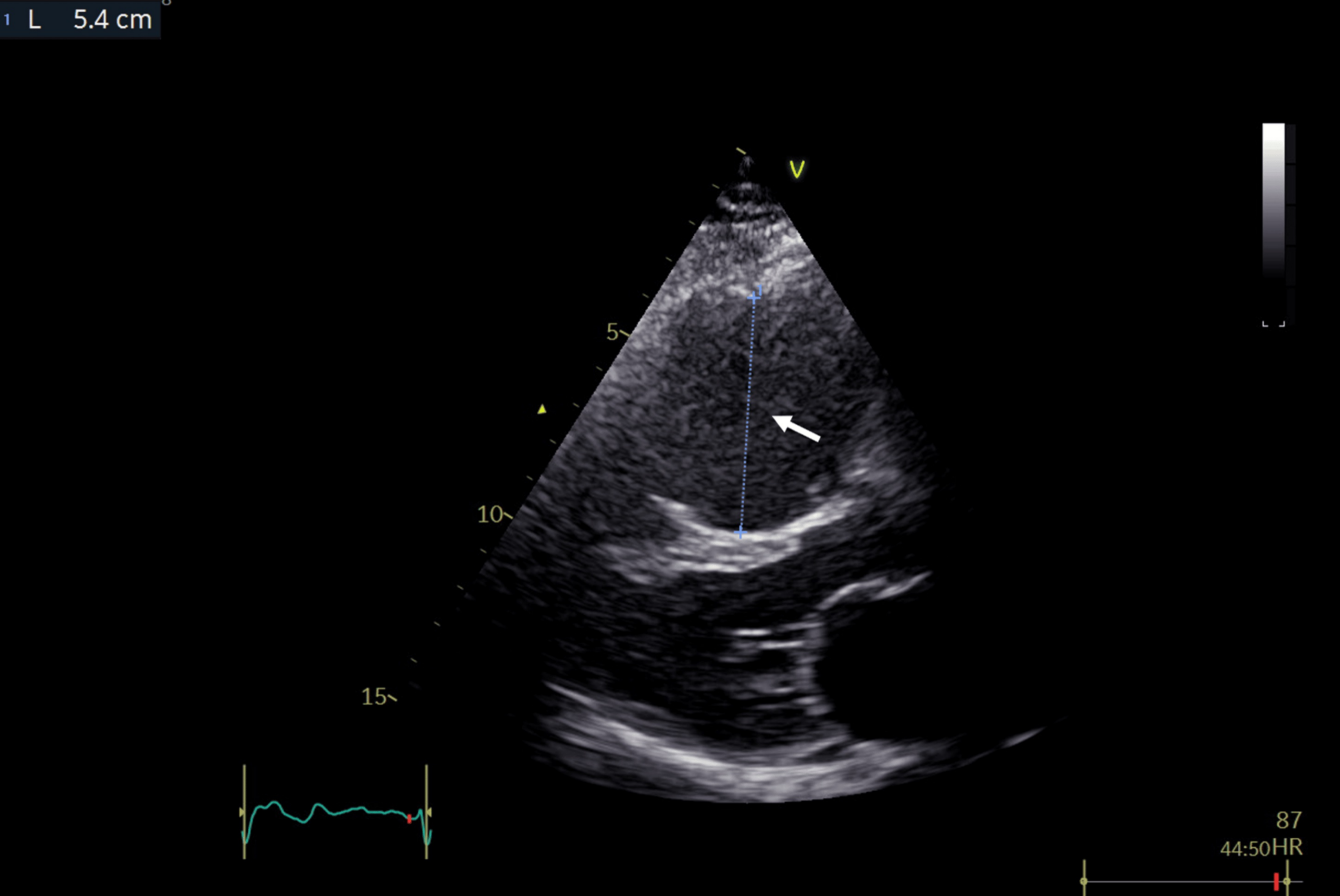
Transthoracic echocardiography (parasternal long-axis view) of Case 2 showing marked right ventricular dilation with an anteroposterior diameter of 54 mm (white arrow)

A 67-year-old woman with well-controlled hypertension presented to the emergency department with palpitations. She had no other significant medical history and had previously had three uncomplicated vaginal deliveries. Electrocardiography revealed atrial fibrillation with rapid ventricular response, hemodynamically well tolerated. TTE revealed a large ostium secundum ASD measuring 45 × 35 × 39 mm, with marked dilation of the right-sided cardiac chambers and severe pulmonary hypertension. Color Doppler quantification demonstrated a significant left-to-right shunt with a Qp/Qs ratio of 2.5. Right heart catheterization was indicated to assess pulmonary vascular resistance and determine eligibility for closure. However, the patient declined further investigation and invasive procedures, opting for conservative management given her age and personal preference (Figures 2-3).

Case 3

**Figure 4:**
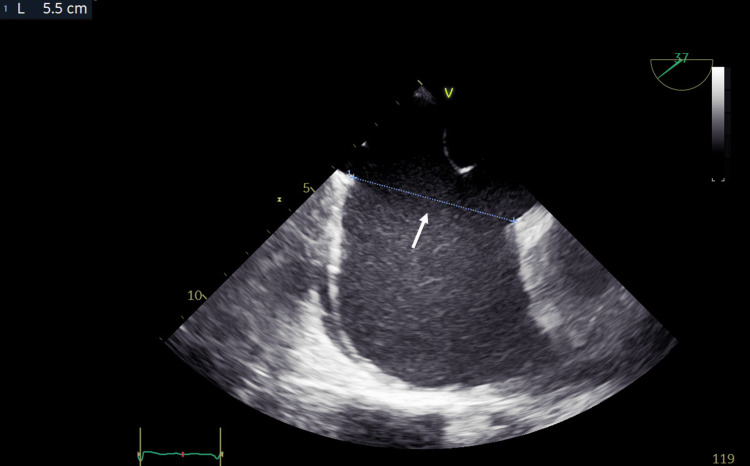
Transesophageal echocardiography (bicaval view) of Case 3 showing a large ostium secundum atrial septal defect (ASD) measuring 55 mm with deficient septal rims (white arrow), precluding percutaneous closure and leading to surgical referral.

A 53-year-old patient with no cardiovascular risk factors was referred for TTE as part of the evaluation of exertional dyspnea. TTE revealed a large ostium secundum ASD measuring 55 × 42 × 40 mm with deficient septal rims and right ventricular dilation. Color Doppler imaging showed a predominant left-to-right shunt ( QP/QS=3) with high probability of pulmonary hypertension. Right heart catheterization confirmed normal pulmonary vascular resistance (PVR = 2 Wood units), excluding fixed pulmonary hypertension. Given the unfavorable anatomy for percutaneous intervention, surgical closure was recommended after heart team discussion, and the patient was referred for surgical repair (Figure 4).

Case 4

**Figure 5:**
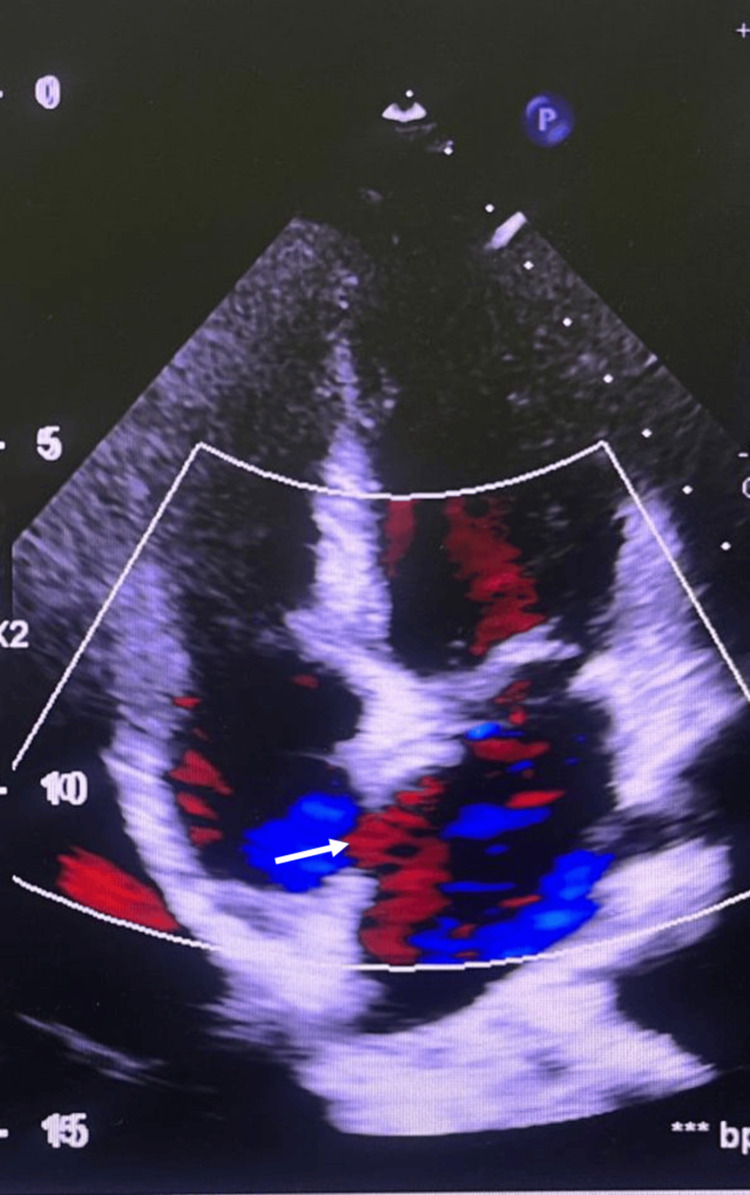
Color Doppler echocardiography (apical four-chamber view) of Case 4 showing a small ostium secundum atrial septal defect (ASD) measuring 8 mm with a minimal left-to-right shunt (white arrow) and no right-sided chamber enlargement, supporting conservative management.

A 50-year-old woman with diabetes mellitus underwent routine TTE as part of standard cardiovascular assessment. She was asymptomatic with no cardiac complaints. Echocardiography incidentally revealed a small ostium secundum ASD measuring 8 mm, associated with a minimal left-to-right shunt (Qp/Qs < 1.5), no right-sided chamber enlargement, and no pulmonary hypertension. Given the absence of hemodynamic repercussions, a conservative strategy with regular echocardiographic follow-up was adopted (Figure 5).

These four cases illustrate the wide clinical and echocardiographic spectrum of ostium secundum ASDs, ranging from early curable forms to late complicated presentations and anatomically or clinically non-eligible cases for closure (Table 1).

**Table 1:** Comparative summary of clinical, echocardiographic, and management characteristics across four cases of ostium secundum atrial septal defect (ASD).

Parameter	Case 1	Case 2	Case 3	Case 4
Age (years)	17	67	53	50
symptoms	Exertional dyspnea	palpitations	Exertional dyspnea	Asymptomatic
Defect size (mm)	20 × 27 × 25	45 × 35 × 39	55 × 42 × 40	8
Qp/Qs	2.0	2.5	3	<1.5
Right ventricle dilatation	Yes	Yes	Yes	No
SPAP (mmHg)	35	55	60	25
Septal rims	Adequate	Adequate	Deficient	-
Management	Percutaneous closure	Conservative	Surgical closure	Conservative

## Discussion

Ostium secundum ASDs most commonly result from a true deficiency of the primum septum and represent the most frequent type of true ASD. The superior and posterior rims of the defect are formed by the secundum septum, the anterior rim by the atrioventricular septum, and the inferior rim by the primum septum and the left venous valve of the inferior vena cava. The morphology of these defects can be highly variable, ranging from elliptical to round shapes. In large ASDs, the primum septum is often markedly reduced or even completely absent. In some cases, remnants of the primum septum may persist, crossing the defect and resulting in multiple openings. The size of ostium secundum ASDs typically ranges from a few millimeters to more than 3 cm in diameter [1].

ASD is the most common congenital heart defect diagnosed in adulthood. Clinical presentation is highly variable and often asymptomatic in children, contributing to underdiagnosis during the early years of life. Symptoms typically arise later in adulthood and are usually related to long-term complications, including pulmonary hypertension, right heart chamber dilation, increased risk of atrial arrhythmias, and thromboembolic events [2,3]. Early diagnosis is therefore crucial to prevent these complications and allow timely intervention before irreversible cardiac remodeling occurs [3,4].

Current European Society of Cardiology (ESC) and American College of Cardiology/American Heart Association (ACC/AHA) guidelines recommend closure in patients with evidence of right ventricular volume overload, regardless of symptom status, provided pulmonary vascular resistance remains below 3 Wood units and there is no significant left ventricular disease [5,6].

This variability is well illustrated by our case series. Case 1 represents a typical early-diagnosed ASD with a Qp/Qs ratio of 2.0 and an SPAP of 35 mmHg, consistent with significant left-to-right shunting without pulmonary hypertension, highlighting the ideal timing for percutaneous intervention as recommended by current ESC and ACC/AHA guidelines [5,6]. In contrast, Case 2 demonstrates an ASD revealed by atrial fibrillation, with a large defect (45 × 35 × 39 mm), a Qp/Qs ratio of 2.4, and an SPAP of 55 mmHg, indicating severe pulmonary hypertension, which precluded closure given the patient’s refusal of further investigation. Case 3 similarly illustrates a late-diagnosed large defect (55 × 42 × 40 mm) with a Qp/Qs ratio of 2.5 and an SPAP of 60 mmHg; despite confirmed reversible pulmonary hypertension (PVR = 2 Wood units), deficient septal rims contraindicated percutaneous closure, leading to surgical referral in accordance with current guidelines [5,6]. Finally, Case 4 shows an incidentally discovered small ASD (8 mm) with a Qp/Qs ratio < 1.5 and an SPAP of 25 mmHg, with no hemodynamic repercussions, for which a conservative surveillance strategy was adopted.

Two-dimensional TTE with color Doppler remains the primary imaging technique for the evaluation of patients with ASDs. It enables detailed assessment of the defect’s size and location, the direction of the interatrial shunt, right heart chamber enlargement, and paradoxical motion of the interventricular septum during diastole, which may indicate significant hemodynamic impact. Spectral Doppler can be used to estimate systolic pulmonary artery pressure from the peak velocity of tricuspid regurgitation, as well as mean and end-diastolic pressures from early and late pulmonary regurgitation velocities. Left-to-right shunt quantification is typically performed using the time-velocity integrals of pulmonary and aortic flows together with their corresponding cross-sectional areas. Additionally, contrast echocardiography using agitated saline mixed with blood can be valuable for detecting intracardiac shunts in patients with suboptimal acoustic windows [2,7].

TEE serves as a complementary imaging modality, typically performed after TTE, offering enhanced visualization of the ASD, particularly in patients with poor transthoracic acoustic windows. TEE is invaluable for detecting associated congenital anomalies, especially anomalous pulmonary venous drainage, and is essential for evaluating the anatomical suitability of patients for percutaneous closure by assessing the size and integrity of the interatrial septal rims. Additionally, it provides critical intra-procedural guidance during catheter-based closure of secundum ASDs in the cardiac catheterization laboratory [1,7].

The use of three-dimensional (3D) TEE significantly enhances the visualization of ASDs. In addition, 3D TEE provides a comprehensive spatial assessment of the defect and its relationships with surrounding cardiac structures and adjacent tissues, thereby improving anatomical understanding and procedural planning. Careful evaluation of the anatomical relationships between the ASD and adjacent structures, including the superior and inferior venae cavae, pulmonary veins, atrioventricular valves, and the coronary sinus, is essential. Adequate surrounding septal rims are required for transcatheter closure; insufficient tissue near the pulmonary veins, atrioventricular valves, or inferior vena cava generally contraindicates the procedure. A rim thickness of at least 5 mm is commonly considered sufficient, while the absence of the aortic rim, although not an absolute contraindication, may increase the risk of device erosion [1].

Intracardiac echocardiography has emerged as a valuable imaging modality for guiding percutaneous structural heart interventions in some centers. Compared with TEE, intracardiac echocardiography provides high-resolution, real-time visualization of intracardiac structures without the need for general anesthesia or esophageal intubation. It allows accurate assessment of the interatrial septum, including defect size and surrounding rims, and enables continuous monitoring during device deployment. In addition, it can be performed by the same interventional operator, eliminating the need for a second operator dedicated to echocardiographic guidance. In the setting of ASD closure, intracardiac echocardiography facilitates optimal device positioning, immediate detection of residual shunts, and early identification of potential complications, making it a safe and effective alternative to TEE guidance in selected patients [8].

Cardiac magnetic resonance is rarely required in the evaluation of ASDs but may provide additional information, particularly for assessing right ventricular volume overload, identifying inferior sinus venosus defects, and quantifying the pulmonary-to-systemic flow ratio (Qp/Qs). Cardiac computed tomography, however, remains the imaging modality of choice for the accurate identification and characterization of anomalous pulmonary venous connections [5].

According to ESC and ACC/AHA guidelines, cardiac catheterization is indicated when non-invasive imaging suggests elevated pulmonary artery pressure, defined by an estimated SPAP >40 mmHg or indirect signs when pulmonary pressure cannot be reliably assessed, to accurately measure pulmonary vascular resistance and confirm eligibility for closure [5,6].

In patients with evidence of right ventricular volume overload and without pulmonary arterial hypertension (no non-invasive signs of elevated pulmonary artery pressure or invasive confirmation of pulmonary vascular resistance <3 Wood units) or left ventricular disease, closure of an ostium secundum ASD is recommended regardless of symptoms [5,6].

According to current guidelines, device closure has become the first-line treatment in anatomically suitable patients, typically those with a stretched defect diameter ≤38 mm and adequate septal rims (≥5 mm, except toward the aorta), representing approximately 80% of cases. This minimally invasive approach is associated with very low mortality, with serious complications occurring in less than 1% of patients, including device erosion and thromboembolic events. Antiplatelet therapy, usually aspirin 75 mg daily, is recommended for at least six months following implantation [5,6,9,10].

Early and sustained improvements in right heart chamber dimensions and pulmonary pressures following percutaneous closure are well documented, underscoring the physiological benefit of eliminating left-to-right shunting [11,12]. Additionally, systematic studies demonstrate a significant decrease in pulmonary hypertension prevalence and mean systolic pulmonary arterial pressure after closure, which supports intervention in patients with elevated pressures when reversible [13].

Surgical repair remains indicated in patients with large defects, deficient rims, multiple defects not amenable to device closure, or associated cardiac anomalies requiring concomitant correction. Surgical closure carries very low mortality (<1% in patients without significant comorbidities) and excellent long-term outcomes, particularly when performed early in life, during childhood or adolescence, and in the absence of pulmonary hypertension [3,5,7].

Our four cases of ostium secundum ASD illustrate the remarkably wide spectrum of clinical and echocardiographic presentations encountered in daily practice. Early diagnosis, as exemplified by Case 1, allows timely percutaneous closure with excellent outcomes before hemodynamic complications arise. In contrast, Cases 2 and 3 highlight the consequences of late diagnosis, including right heart remodeling, pulmonary hypertension, and atrial arrhythmias, which significantly complicate management and may preclude intervention. Case 4 underscores the importance of regular surveillance in hemodynamically insignificant defects. Taken together, these cases confirm that patient age, defect size and morphology, septal rim adequacy, and pulmonary hemodynamics are the key determinants of therapeutic strategy. A patient-centered, anatomy- and physiology-driven approach, supported by multimodality echocardiographic imaging, is essential to optimize outcomes in ostium secundum ASD management.

## Conclusions

Ostium secundum ASDs in adults present a wide clinical and echocardiographic spectrum, from incidental findings to complex cases with right heart remodeling and pulmonary hypertension. Individualized evaluation using transthoracic echocardiography, transesophageal echocardiography, 3D imaging, and intracardiac echocardiography is essential to guide optimal management. Percutaneous closure is safe and effective in anatomically suitable patients, whereas surgical repair remains necessary for complex defects. Early diagnosis and a patient-centered, anatomy-driven approach are crucial to prevent complications and achieve optimal outcomes.
